# Psycho-emotional impact on oncology staff in Abidjan (Côte d'Ivoire): a cross-sectional study

**DOI:** 10.3332/ecancer.2026.2072

**Published:** 2026-02-03

**Authors:** Pétiori G Laurence Touré, Stéphane Ipou, Ismael Kamara, Brahim Samuel Traoré, Bitty Adde Odo, Dion Aristide Gonce, Malan N’Guessan Prosper Mebiala, Gnonsian Estelle Gahie, Etobo Innocent Ahounou, Aka Rita, Kouamé Konan Yvon Kouassi, Moctar Touré, Innocent Adoubi, Gustave Koffi, Jean Marie Yeo Tenena, Roger Charles Joseph Delafosse

**Affiliations:** 1 Faculty of Medical Sciences, Felix Houphouët-Boigny University of Abidjan/Treichville University Hospital of Abidjan, Côte d’Ivoire, West Africa; 2 Faculty of Medical Sciences, Felix Houphouët-Boigny University of Abidjan/National Institute of Public Health of Abidjan, Côte d’Ivoire, West Africa; 3 Faculty of Medical Sciences, Felix Houphouët-Boigny University of Abidjan/Yopougon University Hospital of Abidjan, Côte d’Ivoire, West Africa

**Keywords:** cancer, psycho-oncology, psycho-emotional experience, healthcare workers, Abidjan

## Abstract

**Background:**

The mental health of oncology staff is often impacted by the suffering of patients, in the face of which they frequently feel powerless. The objective of this study was to examine the psycho-emotional experiences of medical and paramedical staff in the oncology and onco-hematology departments of the Treichville and Yopougon University Hospitals.

**Methods:**

We conducted a prospective, descriptive, bicentric study from January 2021 to May 2022 involving 73 healthcare workers recruited through exhaustive sampling. The Hamilton Anxiety Rating Scale was used to assess anxiety among the participants.

**Results:**

Our results revealed that nearly three-quarters of the study participants (73%) had between 1 and 5 years of professional experience and were mostly physicians (72%) or nurses (25%). Symptoms of psychological trauma were observed, particularly avoidance behaviours (46%) and traumatic re-experiencing (46%). A consistently sad mood was reported by 71% of the caregivers. Frequent psychosomatic complaints such as headaches (42%) and epigastric pain (35%) were also noted. Severe anxiety was found in 29% of participants. A shift in personal values was observed, including an increase in religious practices, which rose from 19% to 30%. Finally, 72% of caregivers expressed a desire for psycho-oncological support.

**Conclusion:**

Healthcare personnel in onco-hematology, who care for seriously ill patients often nearing the end of life, are regularly affected on a psycho-emotional level, highlighting the need to establish dedicated psycho-emotional support for this workforce.

## Introduction

The scope of healthcare professionals’ roles in oncology, once focused primarily on end-of-life care, has progressively expanded to include rehabilitation and reintegration of cancer patients. Nevertheless, in the collective imagination, cancer is still often perceived as an incurable disease, inexorably associated with death [1].

Cancer affects not only the individual diagnosed but also profoundly disrupts the family and close relatives, who are confronted with fear, sadness and the alternation of hope and despair [2, 3]. While it was once assumed that healthcare workers were equipped to face their patients’ suffering, the reality in oncology reveals a complex context, characterized by constant stress and daily ethical dilemmas [4]. Several studies have highlighted the psychological impact of such exposure: Lee *et al* [5] reported that oncology nurses experience significant difficulties in coping with the psychological burden of patient death, an experience that influences their end-of-life care practices both negatively and positively. Another study showed that healthcare workers, particularly physicians, may experience intense emotional reactions likely to affect their clinical behaviour, the quality of care delivered, as well as their personal lives [6].

In Côte d’Ivoire, this situation is compounded by the lack of infrastructure and specialized psycho-oncology services, which limit the availability of institutionalized psychological support for healthcare providers. In this context, the mental health of oncology and hematology staff, confronted daily with patient suffering and death, remains a pressing yet underexplored issue.

We therefore conducted this study to explore the psycho-emotional experiences of healthcare personnel working in oncology and hematology departments in Abidjan.

## Methodology

This was a prospective, cross-sectional, bicentric descriptive study conducted in the Oncology Department of Treichville University Hospital and the Hematology Department of Yopougon University Hospital. These departments provide care for patients with hematologic (blood) cancers in the hematology unit and other types of cancer in the oncology unit.

The study was conducted from 1 January 2021, to 31 May 2022 (17 months). We included all healthcare personnel working in these two departments, regardless of their profession (physicians, nurses, nurse assistants, psychologists), with a minimum of 6 months of experience, who were actively on duty at the time of the survey and who gave informed consent to participate. Exclusion criteria were as follows: healthcare workers on medical leave, maternity leave or any other type of leave at the time of the study. Personnel with less than 6 months of professional experience, those who declined to participate and administrative staff in these departments were also excluded.

Sampling was conducted through exhaustive recruitment, meaning that all eligible participants present at the time of data collection were included, without random selection or drawing. Data were collected using a standardized survey form completed during a face-to-face interview with each participant. The Hamilton Anxiety Rating Scale (HAM-A) was used to assess anxiety.

Data were coded and entered in Excel, then transferred to SPSS version 21 for analysis.

Only descriptive statistics were applied, including frequencies, percentages, measures of central tendency and dispersion (mean and standard deviation). Inferential statistics were not used, as the study design was primarily descriptive and exploratory, with a relatively small sample size that did not allow for robust hypothesis testing.

Authorization to conduct the study was obtained from the management of the university hospitals. Participation was voluntary and based on informed consent. Each participant was informed in advance about the study’s objectives, the confidentiality of the data collected and the voluntary nature of their participation, including the right to withdraw at any time without justification, even after initially consenting. Participant anonymity and the confidentiality of all collected data were strictly maintained throughout the study.

## Results

A total of 73 participants met our inclusion criteria.

### Sociodemographic characteristics

The average age of participants was 32.7 years (±5) and 64% were male. Physicians represented 72% of the sample and 73% had 1–5 years of professional experience. A summary of the sociodemographic characteristics is provided in Table 1.

### Psycho-emotional characteristics

Sadness was reported by 71% of participants, and 87% felt helplessness when caring for end-of-life patients. Trauma-related symptoms included avoidance behaviours (46%), re-experiencing (46%) and nervousness (36%). Somatic symptoms were common: headaches (42%) and epigastric pain (35%), most occurring during work hours (68%). Anxiety assessed via HAM-A showed pathological or abnormal levels in 41%, with 29% severe (Table 2).

### Value shifts

Before working in oncology, professional success was the top priority (84%), followed by family success (58%) and financial success (45%). Since beginning in cancer care, emphasis decreased on professional success (64%), family harmony (40%) and financial success (34%), while 30% reported increased religious practice (Table 3).

Most participants (72%) expressed a need for psycho-oncological support.

## Discussion

This study provides valuable insight into the psycho-emotional experiences of healthcare personnel working in oncology and hematology at the Treichville and Yopougon University Hospitals in Côte d’Ivoire. It highlights a significant prevalence of psychological distress among caregivers in these settings.

However, several limitations should be acknowledged. First, the cross-sectional design and relatively small sample size (*n* = 73) limited the analysis to descriptive statistics and prevented causal inference. The study, therefore, aimed to provide an initial situational overview to guide future research using larger and more analytically robust designs. Second, the use of face-to-face interviews may have introduced social desirability bias, potentially leading to an underestimation of anxiety levels. Finally, the relatively small sample size limits the generalizability of the findings to all healthcare workers involved in cancer care.

Despite these limitations, this study is among the first of its kind in the Ivorian oncology context and provides a foundation for future research, particularly larger-scale longitudinal or qualitative studies.

### Sociodemographic profile

The average age in our study is comparable to that reported by Lee *et al* [5], suggesting that many healthcare workers are in the early middle stages of their careers, with professional experience still developing. However, we observed a female predominance, a trend also confirmed by Guariglia *et al* [4].

While we observed a predominance of physicians (72%), other studies often report a greater representation of nursing staff [7, 8]. Nurses typically have more sustained contact with patients and may therefore be more vulnerable to emotional strain. In our context, the predominance of physicians may be because these departments serve as regional training centers for young specialists, and due to the underrepresentation of paramedical professionals in the national oncology workforce.

### Psycho-emotional experiences

Healthcare workers, through their close and daily interactions with patients, inevitably share in their psychological and emotional experiences. In our study, nearly half of the participants (46%) exhibited signs of post-traumatic stress disorder, including avoidance behaviours toward certain patients with particularly distressing clinical presentations, as well as the re-experiencing of patient images, scenes of agony or extreme suffering (46%). According to Phaneuf [9], throughout the caregiving process, healthcare professionals often develop emotional attachment to their patients, rooted in their humanity and vulnerability. This emotional closeness exposes them to a high risk of compassion fatigue, defined as a debilitating exhaustion resulting from repeated empathic responses to the pain and suffering of others, and often associated with burnout and vicarious trauma [9]. Several factors likely contribute to this emotional exhaustion, including difficulties in managing daily stress across all stages of cancer care, starting with the diagnosis, through relapses and recurrences and up to end-of-life support [10]. This is supported by a Moroccan study among oncology professionals, which reported high rates of emotional exhaustion (81.4%) and depersonalization (79.7%) [11]. Similarly, the study by Duarte and Pinto-Gouveia [12] emphasized the key role of psychological factors in the development of burnout (29%) and compassion fatigue (18%) among oncology nurses. It highlighted how prolonged exposure to patient suffering, combined with intense emotional involvement, can lead to significant psychological imbalance, especially when coping mechanisms are inadequate. Moreover, the bitterness of witnessing patients' lives slip away, and the inevitable sadness of failing in what they perceive as their core mission (healing and preserving life), can give rise to feelings of guilt, helplessness in the face of cancer and even self-doubt regarding their clinical competence. In our study, more than three-quarters of caregivers expressed a sense of helplessness and 71% reported feelings of sadness when facing a patient's death or agony. These findings are consistent with those from other settings. For example, Austin *et al* [13] found that healthcare workers often experience high levels of emotional distress when compelled to provide care perceived as ineffective.

In cancer care, caregivers often identify with their patients as a means to deliver high-quality care. However, this identification can foster emotional attachment, explaining why they are often deeply affected by a patient’s death [7]. For some healthcare professionals, cancer care becomes a constraint that challenges their professional positioning [14]. Oncologists, in particular, express frustration over their inability to fully manage their patients' care in the face of therapeutic failure. This sense of helplessness often leads to resignation and is accompanied by feelings of guilt [10].

### Anxiety and somatic symptoms

Added to this is the inherent complexity of working in oncology units, especially when supporting patients in distress or severe pain. Such conditions cause physical exhaustion that quickly escalates into psychological overload, resulting in intense emotional stress that can develop into pathological anxiety [15]. This may help explain the relatively high proportion of caregivers in our study presenting with severe or pathological anxiety (29%), which inevitably impacts work organization and the quality of care provided. This situation has also been documented in Spain, where a study among oncology nurses reported that 20% experienced burnout and 37% secondary traumatic stress due to constant exposure to patient suffering. These disorders were associated with high levels of both state and trait anxiety, contributing to demotivation, the desire to leave the unit and even to exit the profession altogether [16]. Furthermore, Lissandre *et al* [17] demonstrated that emotional exhaustion contributes to a decline in the quality of life of healthcare professionals in oncology and hematology, due to ineffective workload management and communication difficulties.

Most caregivers in our study reported somatic symptoms, primarily headaches (42%) and epigastric pain (35%), with 68% indicating these symptoms occurred predominantly in the workplace. These manifestations are often part of a broader context of emotional stress and represent physical expressions of anxiety, which can take many forms. Our findings are consistent with literature linking emotional exhaustion to various somatic symptoms, including general malaise, gastrointestinal issues, headaches, chronic fatigue, respiratory difficulties and even serious injuries and increased mortality risk before the age of 45 [18]. This confirms that emotional distress in oncology extends beyond psychological suffering, manifesting physically as well.

### Value shifts and coping strategies

Before their engagement in oncology, the dominant values among our respondents were professional achievement (84%) and family harmony (58%). However, these priorities have declined since they began practicing in this field, now concerning only 64% and 40% respectively, in favour of a stronger emphasis on spirituality, with faith increasing from 19% to 30%. This shift appears to be linked to repeated exposure to suffering, death and the limits of medicine. Faced with a sense of therapeutic helplessness, caregivers find in faith a psychological and spiritual resource that helps them give meaning to end-of-life care, better manage stress and ease the emotional burden of repeated patient deaths. Spiritual realignment thus emerges as both a protective psychological mechanism and a factor of resilience in a particularly demanding professional environment. Rushton *et al* [19] confirm this dynamic by showing that spiritual support strengthens caregivers’ resilience in high-intensity contexts, while reducing emotional exhaustion and depersonalization. Similarly, a Spanish study reported that oncology professionals resort to spirituality as a coping strategy to deal with the emotional demands of daily practice and the persistent suffering of patients [20].

### Support needs and implications

Finally, 72% of respondents expressed a need for psychological support. In this regard, support groups appear to be a relevant intervention, having demonstrated their effectiveness in improving the psychological well-being of healthcare professionals [21]. Vanbockstael *et al* [15], in their study on burnout among medical residents, also highlighted the value of peer discussion groups, which allow participants to share experiences, gain perspective and better cope with the stress of caring for suffering cancer patients.

### Implications for policy and practice

In resource-limited settings such as Côte d’Ivoire, where psycho-oncology infrastructure remains scarce, these findings highlight the urgency of integrating caregiver mental health into oncology services. Practical measures include implementing structured support groups, offering systematic training in stress and emotion management and creating referral pathways for specialized mental health care. At the policy level, investments in psycho-oncology units and caregiver well-being programs would strengthen resilience among staff and ultimately improve the quality of cancer care.

## Conclusion

Healthcare workers in oncology and hematology face heavy emotional demands, with frequent manifestations of sadness, helplessness, anxiety and somatization, particularly among younger physicians. These experiences are often accompanied by shifts in personal values, including decreased emphasis on professional and social success and increased reliance on spirituality as a coping mechanism.

Beyond individual consequences, these findings underline the importance of institutional and policy-level initiatives to protect caregivers’ mental health and sustain the quality of cancer care. Future research should build on this work with larger, more diverse samples and analytical designs to better identify risk and resilience factors.

## Conflicts of interest

The authors declare that they have no conflicts of interest.

## Funding

This research received no specific grant from any funding agency in the public, commercial or not-for-profit sectors.

## Data availability

The data utilized in this study can be provided by the authors upon request.

## Figures and Tables

**Table 1.** Sociodemographic characteristics of healthcare workers.

Variables	Frequency (*n*)	Percentage (%)
Age		
≤30 years	15	21
31–40 years	39	57
>40 years	19	26
Gender		
Male	21	43
Female	42	57
Profession		
Physician	53	73
Nurse	18	25
Health assistant	2	2
Years of experience		
1–5 years	53	73
>5 years	20	27

**Table 2.** Characteristics of the psycho-emotional experience of healthcare workers.

Variables	Frequency (*n*)	Percentage (%)
Lifestyle		
Alcohol consumption	34	46
Perception of the patient by the staff		
In need of care	72	99
Condemned	15	21
Post-traumatic stress symptoms		
Avoidance	34	46
Flashbacks	34	46
Nervousness	26	36
Feelings toward the patient		
Helplessness	64	87
Sadness	55	75
Experience during the patient's agony		
Sadness	52	71
Somatic symptoms		
Headaches	31	42
Epigastric pain	26	35
Location of somatic symptoms		
At the hospital	50	68
Outside the hospital	23	31
HAM-A scale score		
Normal anxiety	47	64
Severe anxiety	21	29

**Table 3.** Characteristics related to value shifts among participants before and after working in oncology.

Variables	Before *n* (%)	After *n* (%)
Professional achievement	61 (84)	47 (64)
Financial success	33 (45)	24 (34)
Family harmony and social cohesion	42 (58)	29 (40)
Life goals	24 (33)	19 (26)
Spirituality	14 (19)	22 (30)
